# BMC Nursing reviewer acknowledgement 2014

**DOI:** 10.1186/s12912-015-0057-y

**Published:** 2015-02-18

**Authors:** Catia Cornacchia

**Affiliations:** BioMed Central, Floor 6, 236 Gray’s Inn Road, London, WC1X 8HB UK

**Masoud Amiri**

Netherlands

**Ergul Aslan**

Turkey

**Mohammadkarim Bahadori**

Iran

**James Balmford**

Australia

**Jean Barry**

Switzerland

**Sandra Bassendowski**

Canada

**Linda Baumann**

USA

**Dimitri Beeckman**

Belgium

**Carol Bennett**

Canada

**Linda Beuscher**

USA

**Melanie Bish**

Australia

**Joyce Black**

USA

**Nicole Blay**

Australia

**Sandy Braaf**

Australia

**Robyn Cant**

Australia

**Pierre Cardinal**

Canada

**Roy Carr-Hill**

UK

**Kristin Carson**

Australia

**Gianluca Catania**

Italy

**Ching-Min Chen**

Taiwan

**James Church**

USA

**Pamela Clarke**

USA

**Simon Cooper**

Australia

**Debra Creedy**

Australia

**Greta Cummings**

Canada

**Laura Damschroder**

USA

**Daniela D'Angelo**

Italy

**Ulrike Dapp**

Germany

**Rene Day**

Canada

**Maura Dowling**

Ireland

**Marc Du Bois**

Belgium

**Renee Du Toit**

South Africa

**Nuhad Dumit**

Lebanon

**Nancy Dunton**

USA

**Susan Dyess**

USA

**Georgios Efstathiou**

Cyprus

**Michaela Eikermann**

Germany

**Özgül Erol**

Turkey

**Mohammad Esmaeili Abdar**

Iran

**Mohammad Esmaeilpour Bandboni**

Iran

**Ali Fakhr-Movahedi**

Iran

**Deirdre Fetherstonhaugh**

Australia

**Helen Franks**

UK

**Hiroki Fukahori**

Japan

**Martha Funnell**

USA

**Peter Gallagher**

Nigeria

**Robyn Gallagher**

Australia

**Laurent Gerbaud**

France

**Charlie Goldsmith**

Canada

**Helen Goodman**

UK

**Laura Goodwin**

UK

**Sue Green**

UK

**Rosane Griep**

Brazil

**Ruud Halfens**

Netherlands

**Wendy Hall**

Canada

**Catherine Hankey**

UK

**Tove Hanssen**

Norway

**Ian Harris**

Australia

**Gørill Haugan**

Norway

**Cornelia Heinze**

Germany

**Amanda Henderson**

Australia

**Ingela Henoch**

Sweden

**Mary Hickson**

UK

**Karen Hoare**

New Zealand

**Nancy Hogan**

USA

**Wiliam Holzemer**

USA

**Doris Howell**

Canada

**Suh-Ing Hsieh**

Taiwan

**Robbert Huijsman**

Netherlands

**Marie Hutchinson**

Australia

**Jeanette Ignacio**

Singapore

**Sedigheh Iranmanesh**

Iran

**David Jolley**

UK

**Chi-Wen Juan**

Taiwan

**Sharon Kaasalainen**

Canada

**Eulalia Kahwa**

Jamaica

**Mari Kangasniemi**

Finland

**Maria Karanikola**

Cyprus

**Judith Karshmer**

USA

**Sultan Kav**

Turkey

**Daniel Kelly**

UK

**Robin Kennedy**

UK

**Carole Kenner**

USA

**Michael Kerr**

Canada

**Sharon Kinney**

Australia

**Helen Kirkpatrick**

Canada

**Ruth M. Kleinpell**

USA

**Serena Koh**

Singapore

**Rachel Kornhaber**

Australia

**Veronica Lambert**

Ireland

**Kylie Lange**

Australia

**Heather Laschinger**

Canada

**Katherine Laux Kaiser**

USA

**I-Chuan Li**

Taiwan

**Sok Ying Liaw**

Singapore

**Violeta Lopez**

Singapore

**Marta Losa Iglesias**

Spain

**Andrew Lovell**

UK

**Lee-Fay Low**

Australia

**Tobias Luck**

Germany

**Marinha Sofia Macedo**

Saudi Arabia

**Sandra Mackey**

Australia

**Salvatore R Maddi**

USA

**Ganga Mahat**

USA

**Moira Maley**

Australia

**Sumaira Malik**

UK

**Anastasia Mallidou**

Canada

**Judy Mannix**

Australia

**Anna Marchetti**

Italy

**Wanda Martin**

Canada

**Randi Martinsen**

Norway

**Anne Matthews**

Ireland

**Neda Mehrdad**

Iran

**Anastasios Merkouris**

Cyprus

**Patricia R Messmer**

USA

**Patriek Mistiaen**

Netherlands

**Yoshifumi Miyazaki**

Japan

**Ralph Möhler**

Germany

**Zena Moore**

Ireland

**Wendy Moyle**

Australia

**Terrence Murphy**

USA

**Teri Murray**

USA

**Noureddin Nakhostin Ansari**

Iran

**Heather Newton**

UK

**Patricia Nicholson**

Australia

**Alireza Nikbakht**

Nasrabadi Iran

**Nicola North**

New Zealand

**Robert Olson**

Canada

**Linda O'Mara**

Canada

**Daniel O'Neill**

USA

**Eme Owoaje**

Nigeria

**Wolter Paans**

Netherlands

**Savvas Papacostas**

Cyprus

**Evridiki Papastavrou**

Cyprus

**Pammla Petrucka**

Tanzania

**Kathryn Pfaff**

Canada

**Jane Phillips**

Australia

**Barbara Polivka**

USA

**Davina Porock**

UK

**Janet Primomo**

USA

**Jamal Qaddumi**

Palestinian Territory

**Hossein Rafiei**

Iran

**Paul Ratanasiripong**

USA

**Michael Relf**

USA

**Anne-Sophie Rigaud**

France

**Jacqueline Ross**

USA

**Elizeus Rutebemberwa**

Uganda

**Ali Saleh**

Jordan

**Pavlos Sarafis**

Greece

**Stephanie Schim**

USA

**P Anne Scott**

Ireland

**Walter Sermeus**

Belgium

**Nick Sevdalis**

UK

**Abeer Shaheen**

Jordan

**Caroline Shuldham**

UK

**Maja Söderbäck**

Sweden

**Panayota Sourtzi**

Greece

**Annelie Sundler Johansson**

Sweden

**Susan Swider**

USA

**Miyuki Takase**

Japan

**Kirsti Torjuul**

Norway

**Hsiu-Min Tsai**

Taiwan

**Mimi Tse**

Hong Kong

**Sharon Tucker**

USA

**Mojtaba Vaismoradi**

UK

**Peter Hj Van Der Voort**

Netherlands

**Anneloes Van Staa**

Netherlands

**James Vardaman**

USA

**Marianne Wallis**

Australia

**Zhen Wang**

USA

**Caryn West**

Australia

**Lesley Wilkes**

Australia

**Emma Wilkinson**

UK

**Lilian Wong**

Singapore

**Bobbi Jo Yarborough**

USA

**Kourosh Zarea**

Iran

